# Incidence of and Characteristics Associated With Long-term Benzodiazepine Use in Finland

**DOI:** 10.1001/jamanetworkopen.2020.19029

**Published:** 2020-10-29

**Authors:** Heidi Taipale, Hanna Särkilä, Antti Tanskanen, Terhi Kurko, Tero Taiminen, Jari Tiihonen, Reijo Sund, Annamari Tuulio-Henriksson, Leena Saastamoinen, Jarmo Hietala

**Affiliations:** 1 Department of Forensic Psychiatry, University of Eastern Finland, Niuvanniemi Hospital, Kuopio; 2 Department of Clinical Neuroscience, Karolinska Institutet, Stockholm, Sweden; 3 University of Eastern Finland School of Pharmacy, Kuopio; 4 Turku University Hospital, Department of Psychiatry, University of Turku, Turku, Finland; 5 City of Turku Welfare Division, Turku City Hospital, Turku, Finland; 6 Research Unit, The Social Insurance Institution, Helsinki, Finland; 7 Center for Psychiatry Research, Stockholm City Council, Stockholm, Sweden; 8 University of Eastern Finland School of Medicine, Kuopio; 9 Department of Psychology and Logopedics, University of Helsinki, Helsinki, Finland

## Abstract

**Importance:**

The proportion of patients who develop long-term benzodiazepine use remains controversial, as do the length of time before long-term use develops and the factors associated with long-term use.

**Objective:**

To investigate the incidence of long-term benzodiazepine and related drug (BZDR) use and factors associated with the development of long-term use implementing a follow-up design with new BZDR users.

**Design, Setting, and Participants:**

This population-based cohort study used a nationwide cohort of 129 732 new BZDR users in Finland. New users of BZDRs aged 18 years or older were identified from the prescription register maintained by the Social Insurance Institution of Finland as individuals who initiated BZDR use during 2006 and had not used BZDRs from 2004 to 2005. The follow-up continued until death, long-term hospitalization, a gap of 2 years in BZDR use, or December 31, 2015. The population was analyzed according to age at treatment initiation, categorized into younger (<65 years) and older (≥65 years) subcohorts. Analyses were conducted from May 2019 to February 2020.

**Exposures:**

Use of BZDRs, modeled from register-based data using the PRE2DUP (from prescriptions to drug use periods) method.

**Main Outcomes and Measures:**

Long-term BZDR use, defined as continuous use of 180 days or longer, and factors associated with long-term vs short-term use, compared using Cox proportional hazards models.

**Results:**

Among the 129 732 incident BZDR users, the mean (SD) age was 52.6 (17.7) years, and 78 017 (60.1%) individuals were women. During the follow-up period, 51 099 BZDR users (39.4%) became long-term users. Long-term treatment was more common in the older subcohort (19 103 individuals [54.5%]) than the younger subcohort (31 996 individuals [33.8%]). At 6 months, 28 586 individuals (22.0%) had become long-term users: 11 805 (33.7%) in the older subcohort and 16 781 (17.7%) in the younger subcohort. The largest proportions of initiators who became long-term users were those persons who initiated treatment with nitrazepam (76.4%; 95% CI, 73.6%-79.1%), temazepam (63.9%; 95% CI, 62.9%-65.0%), lorazepam (62.4%; 95% CI, 59.7%-65.1%), or clonazepam (57.5%; 95% CI, 55.9%-59.2%). Factors associated with the development of long-term use included male sex, older age, receipt of social benefits, psychiatric comorbidities, and substance abuse.

**Conclusions and Relevance:**

The findings of this population-based cohort study conducted in Finland suggest that the incidence of subsequent long-term BZDR use in individuals who initiate use of BZDRs is high, especially among older persons, and that the specific BZDR used initially is associated with the development of long-term BZDR use and should be carefully considered when prescribing BZDRs. The observed factors that appear to be associated with development of long-term BZDR use also should be considered in clinical decision-making when starting and monitoring BZDR treatment.

## Key Points

**Questions:**

What is the incidence of long-term use of benzodiazepines and related drugs (BZDRs) in persons who start new BZDR treatment, and what factors are associated with the development of long-term use?

**Findings:**

In a cohort study of 129 732 new BZDR users in Finland, 34% of working-aged persons and 55% of older persons developed long-term use. Higher rates of long-term use were associated with specific drugs, namely, nitrazepam, temazepam, lorazepam, and clonazepam.

**Meaning:**

Despite guidelines and recommendations, BZDRs are still prescribed frequently for long-term treatment, especially in older persons, and the initial choice of a specific BZDR is associated with development of long-term BZDR use.

## Teaser

This cohort study uses national longitudinal data from the Social Insurance Institution of Finland to investigate the incidence of long-term use of benzodiazepines and related drugs among new users and factors associated with development of long-term use.

## Introduction

The rationale for long-term use of benzodiazepines and related drugs (BZDRs) has long been a subject that has divided physicians all over the world.^1,2,3^ The main concerns about benzodiazepine use are related to development of tolerance and dependence on regular use as well as adverse events, especially in older individuals.^4,5^ Consequently, several guidelines and recommendations for BZDR prescribing have been published over the years, emphasizing that duration of use should be limited to the short term (4-12 weeks).^6,7,8,9,10,11,12,13^ Some authors also suggest that BZDR benefits may persist, at least in specific patient groups,^14,15,16,17^ and that the risk-benefit ratio of long-term use (eg, in anxiety disorders) has not been properly studied in comparison with alternative pharmacotherapeutic approaches such as the administration of selective serotonin reuptake inhibitors.^18,19^

Despite all the recommendations, a vast amount of literature shows that long-term use is still very common, especially in older patients, and further research on the topic is needed to optimize the use of BZDRs.^20,21^ Among older persons, BZDR use has been associated with cognitive and psychomotor adverse effects and an increased risk of falls,^22^ fractures,^23,24^ and even mortality.^25^ General factors associated with long-term BZDR use, according to previous studies, include sex, comorbid conditions, older age, lower income, and poorer health status.^20,26,27^ Some studies point toward problems focused on specific substances, including alprazolam and clonazepam.^28,29,30^ Concerns have also been raised about the increasing use of benzodiazepine-related drugs, often called Z-drugs, specifically, zolpidem.^28,30,31^

However, more information is needed in clinical decision-making when initiating the use of BZDRs, renewing prescriptions, and attempting discontinuation of use. The objective of this study was to investigate the incidence of long-term BZDR use and factors associated with development of long-term use.

## Methods

This cohort study used data from national, longitudinal, population-based registers in Finland. These registers are linkable through unique personal identity codes that are assigned for all residents, ie, all residents potentially could have been included in the study. The study population was identified from the prescription register maintained by the Social Insurance Institution of Finland. We used data from January 1, 2004, to December 31, 2015, on all reimbursed prescription drug purchases for all residents, categorized according to Anatomical Therapeutic Chemical (ATC) classification codes. Data were linked from the Care Register for Health Care (January 1, 2004, to December 31, 2015), including dates of hospital admissions, discharges, and diagnoses. Data were also collected from other registers of the Social Insurance Institution of Finland, including the special reimbursement register, which contains data since 1972 on persons granted a special refund for drugs because of diagnosed chronic diseases; a register of persons who receive social benefits (based on basic social assistance, labor market subsidy, basic unemployment allowance, national pension, and study grants); and a register of persons who receive child care benefits (maternity allowance, paternity allowance, parental allowance, and child home care allowance). Data on disability pensions were extracted from the Finnish Centre for Pensions (2004-2015). Pertinent institutional authorities at the Finnish Institute for Health and Welfare, the Social Insurance Institution of Finland, the Finnish Centre for Pensions, and Statistics Finland granted permission for this study. According to Finnish legislation, no ethics committee approval or patient consent is required for deidentified register-based research. This study followed the Strengthening the Reporting of Observational Studies in Epidemiology (STROBE) reporting guideline.

In this study, BZDR use was defined as ATC codes N03AE01 (clonazepam), N05BA, N05CD, N05CF, and N06CA01 (including chlordiazepoxide) (eTable 1 in the Supplement). Duration of continuous BZDR use was defined using the PRE2DUP (from prescriptions to drug use periods) method (eAppendix in the Supplement).^32^

The study identified 132 550 persons 18 years or older who initiated BZDR use during 2006 without BZDR use during 2004 to 2005, and they were defined as incident users (eFigure 1 in the Supplement). Long-term use was defined as continuous use lasting 180 days or longer.^20^ Based on this definition, we excluded 2818 persons who had less than 180 days of follow-up owing to death or long-term (>90 days) hospitalization. Thus, the study cohort included 129 732 persons. The follow-up started at the date of first benzodiazepine purchase (index date) and ended when the definition of long-term use was fulfilled at death, at long-term hospitalization, after 10 years of follow-up, or after 2 years of BZDR nonuse, whichever occurred first. Drug use during hospitalizations is not recorded in the prescription register; thus, exposure status is not known during long hospital care periods. The outcome was 180 days or more of continuous BZDR use, and persons were allowed to have gaps in BZDR use as long as the gap did not exceed 731 days (which corresponds to the length of the washout period for this study). Sensitivity analyses were conducted by restricting the permissible gap to 365 days. Factors associated with long-term BZDR use and exact definitions are provided in eTable 1 in the Supplement.

### Statistical Analyses

Descriptive statistics were used to summarize characteristics of incident BZDR users, divided into 2 subcohorts (<65 years and ≥65 years), and to assess incidence of long-term use. Specific drugs were categorized according to the drug or drugs dispensed at the initiation; polytherapy refers to the dispensing of 2 or more BZDRs at the same time. For specific drugs, the proportion of initiators who became long-term users was calculated with 95% CIs, as was the proportion of long-term users who quickly progressed to long-term use without having gaps in drug use (≥180 days of continuous use after initiation).

Time to initiation of long-term use was described by plotting the cumulative incidence of long-term use with time, considering the competing risks of death and long-term hospitalization and censoring to a 2-year gap. Factors associated with long-term use compared with short-term use were assessed with Cox proportional hazards models, and the results were reported as hazard ratios (HRs) with 95% CIs. Proportional hazards assumptions were confirmed by exploring parallelism of log-negative and log-estimated survival curves. The effect of each variable was adjusted for the effects of all other variables in a full model. Variables with a strong correlation with 1 or more other variables and variables with a cell count of less than 200 were removed. Statistical analysis was conducted using SAS statistical software, version 9.4 (SAS Institute). Data were analyzed from May 2019 to February 2020.

## Results

### Study Population

The study population included all 129 732 incident BZDR users in 2006 in Finland. Age ranged from 18 to 103 years, with a mean (SD) age of 52.6 (17.7) years, and 78 017 individuals (60.1%) were women. In the younger subcohort of 94 674 individuals, the mean (SD) age was 44.2 (12.4) years, the median age was 46 (interquartile range [IQR], 35-55) years, and 55 091 (58.2%) were women. In the older subcohort of 35 058 individuals, the mean (SD) age was 75.2 (6.9) years, the median (IQR) age was 75 (69-80) years, and 22 926 (65.4%) were women.

### Most Common BZDRs Initiated

In both subcohorts, zopiclone was the most common BZDR at the initiation of use; 30 176 (31.9%) of the younger users and 13 875 (39.6%) of the older users initiated treatment with zopiclone. The second, third, and fourth most common substances were zolpidem (21.2%), oxazepam (15.7%), and alprazolam (9.8%) for the younger users and oxazepam (16.2%), temazepam (12.6%), and zolpidem (11.8%) for the older subcohort. Initiation with more than 1 BZDR simultaneously was observed for 2109 individuals (2.2%) in the younger subcohort and 444 individuals (1.3%) in the older subcohort.

### Incidence of Long-term Use

During the study follow-up period (median [IQR], 2.1 [1.0-2.9] years), 51 099 BZDR users (39.4%) became long-term users. In total, 31 996 individuals (33.8%) in the younger subcohort fulfilled the definition for long-term use during the median (IQR) follow-up period of 2.1 (1.7-3.0) years, whereas the corresponding figure for the older subcohort was 19 103 individuals (54.5%) during the median (IQR) follow-up of 2.1 (0.5-2.6) years. Cumulative incidence of long-term use is presented in eFigure 2 in the Supplement. At 6 months, 28 586 individuals (22.0%) had become long-term users, and the proportion was larger among the older subcohort (11 805 individuals [33.7%]) than in the younger subcohort (16 781 [17.7%]).

### Description of Long-term Users

**Figure 1.**
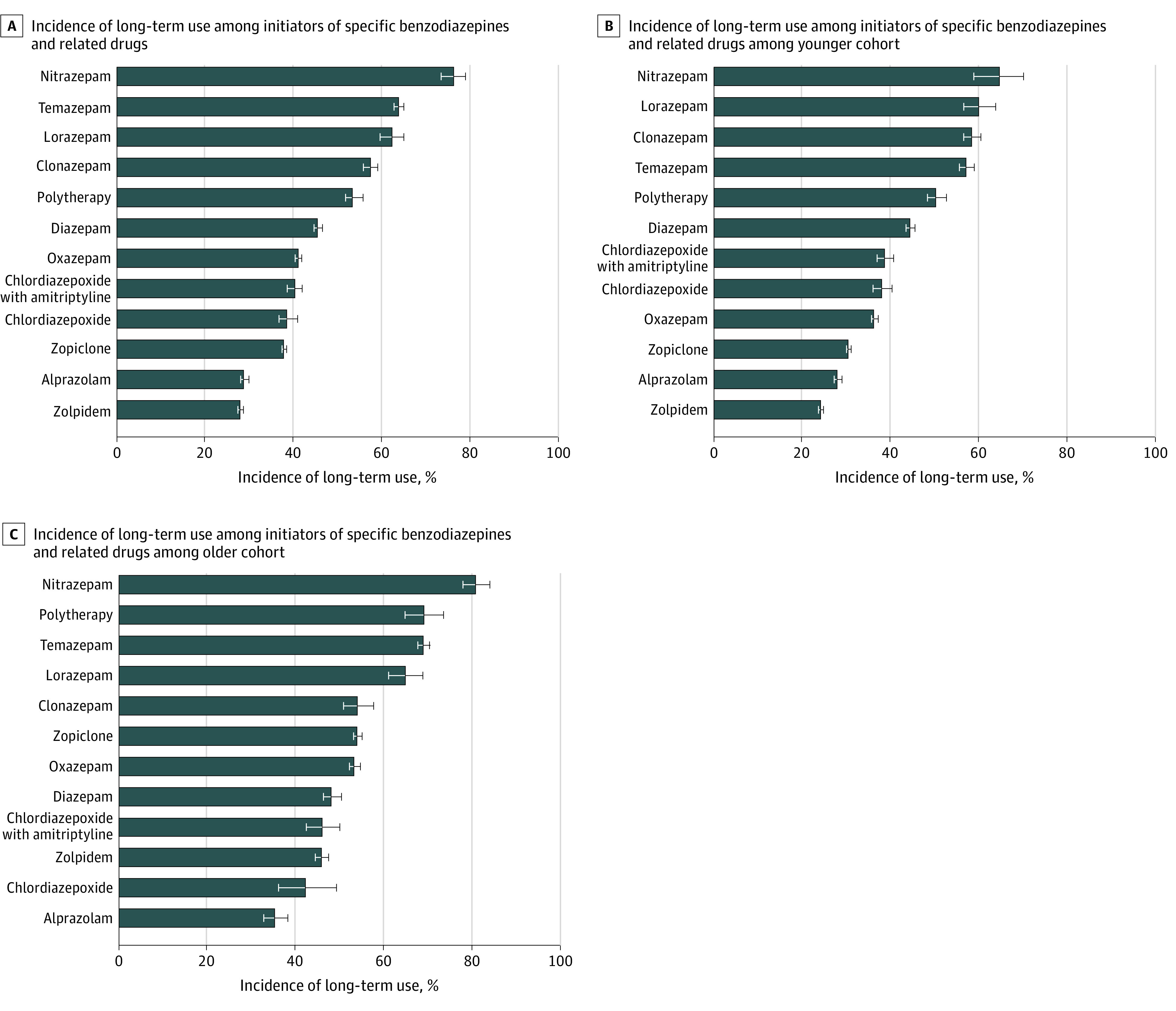
Incidence of Long-term Use Among Initiators of Treatment With Specific Benzodiazepines and Related Drugs During Follow-up Error bars indicate 95% CIs.

Two measures describe the potential for long-term use: the proportion of initiators who become long-term users and the proportion of long-term users who progress to long-term use without gaps in drug use. The largest proportions of initiators who became long-term users were those who initiated treatment with nitrazepam (76.4%; 95% CI, 73.6%-79.1%), temazepam (63.9%; 95% CI, 62.9%-65.0%), lorazepam (62.4%; 95% CI, 59.7%-65.1%), or clonazepam (57.5%; 95% CI, 55.9%-59.2%) (Figure 1). Among those who became long-term users, the largest proportions without gaps in use were those who used lorazepam (84.2%), nitrazepam (83.9%), or clonazepam (80.0%) (Table 1). In terms of both measures, those who initiated with zolpidem had the lowest probability of long-term use; 27.9% became long-term users, and 31.0% of those had no gaps in use. The mean (SD) age of initiators was highest for nitrazepam (70.5 [14.6] years) and temazepam (65.6 [16.2] years) and lowest for alprazolam (43.8 [15.8] years). Extremities of sex distribution were noted for those who initiated treatment with an amitriptyline-chlordiazepoxide combination (71.0% women) and chlordiazepoxide (26.8% women).

**Table 1.** Age and Sex Distribution of Initiators of Specific Drugs and the Proportion of Long-term Users Who Did Not Have Gaps in Drug Use Before Long-term Use Definition Was Fulfilled

Drug	Initiators			Long-term users who did not have gaps in use before long-term use, No. (%)
	No.	Age, mean (SD), y	No. (%) Women	
Lorazepam	1217	59.0 (19.3)	750 (61.6)	640 (84.2)
Nitrazepam	916	70.5 (14.6)	559 (61.0)	587 (83.9)
Clonazepam	3382	51.5 (17.1)	1839 (54.4)	1557 (80.0)
Amitriptyline + chlordiazepoxide	3261	51.0 (15.2)	2314 (71.0)	1039 (79.0)
Temazepam	7820	65.6 (16.2)	4545 (58.1)	3870 (77.4)
Polytherapy	2553	48.2 (17.0)	1284 (50.3)	978 (71.2)
Oxazepam	20 578	52.7 (18.1)	13 111(63.7)	5441 (64.3)
Diazepam	9382	52.2 (17.2)	4980 (53.1)	2733 (63.9)
Alprazolam	10 437	43.8 (15.8)	6374 (61.1)	1527 (50.7)
Chlordiazepoxide	2022	47.7 (13.5)	542 (26.8)	384 (49.1)
Zopiclone	44 051	54.7 (17.5)	26 453 (60.1)	7740 (46.3)
Zolpidem	24 113	48.6 (16.1)	15 266 (63.3)	2090 (31.0)

Univariate associations of characteristics associated with long-term use compared with short-term use are presented in Table 2 (entire cohort) and in eTable 2 (younger subcohort) and eTable 3 (older subcohort) in the Supplement.

**Table 2.** Characteristics of Short-term Compared With Long-term Users of BZDRs in the Cohort of 129 732 Individuals

Characteristic	Users, No. (%)	
	Short-term	Long-term
Age category, y		
18-34	16 892 (21.5)	6439 (12.6)
35-49	22 667 (28.8)	10 907 (21.3)
50-64	23 119 (29.4)	14 650 (28.7)
65-74	8743 (11.1)	8643 (16.9)
75-85	6123 (7.8)	8687 (17.0)
>85	1089 (1.4)	1773 (3.5)
Male sex	29 735 (37.8)	21 980 (43.0)
First dispensed BZDR		
Zopiclone	27 324 (34.8)	16 727 (32.7)
Zolpidem	17 377 (22.1)	6736 (13.2)
Oxazepam	12 110 (15.4)	8468 (16.6)
Alprazolam	7424 (9.4)	3013 (5.9)
Diazepam	5103 (6.5)	4279 (8.4)
Temazepam	2821 (3.6)	4999 (9.8)
Amitriptyline + chlordiazepoxide	1946 (2.5)	1315 (2.6)
Clonazepam	1436 (1.8)	1946 (3.8)
Chlordiazepoxide	1240 (1.6)	782 (1.5)
Polytherapy	1179 (1.5)	1374 (2.7)
Lorazepam	457 (0.6)	760 (1.5)
Nitrazepam	216 (0.3)	700 (1.4)
Other medication use (≤30 d before BZDR treatment initiation)		
Antidepressants	14 804 (18.8)	11 461 (22.4)
Nonopioid analgesics	13 266 (16.9)	9175 (18.0)
Antipsychotics	3128 (4.0)	5453 (10.7)
Muscle relaxants	2376 (3.0)	1558 (3.1)
Opioids	1472 (1.9)	1625 (3.2)
Comorbidities		
Hypertension	11 446 (14.6)	11 860 (23.2)
Asthma or COPD	5396 (6.9)	4245 (8.3)
Coronary artery disease	4865 (6.2)	6121 (12.0)
Cancer	3602 (4.6)	3357 (6.6)
Diabetes	3148 (4.0)	3691 (7.2)
Substance abuse	2733 (3.5)	3254 (6.4)
Hypothyroidism	2050 (2.6)	1671 (3.3)
Rheumatoid arthritis	2011 (2.6)	1598 (3.1)
Schizophrenia	1603 (2.0)	2688 (5.3)
Chronic heart failure	1586 (2.0)	2505 (4.9)
Epilepsy	1126 (1.4)	1040 (2.0)
Alzheimer disease	1084 (1.4)	1725 (3.4)
Stroke	931 (1.2)	1273 (2.5)
Bipolar disorder	687 (0.9)	839 (1.6)
Inflammatory bowel disease	656 (0.8)	380 (0.7)
Parkinson disease	447 (0.6)	682 (1.3)
Multiple sclerosis	221 (0.3)	195 (0.4)
ADHD	112 (0.1)	100 (0.2)
Other factors		
Receipt of social benefits	23 241 (29.6)	23 472 (45.9)
Discharge from hospital ≤2 wk	11 733 (14.9)	9610 (18.8)

Abbreviations: ADHD, attention-deficit/hyperactivity disorder; BZDR, benzodiazepine and related drug; COPD, chronic obstructive pulmonary disease.

### Factors Associated With Long-term Use

**Figure 2.**
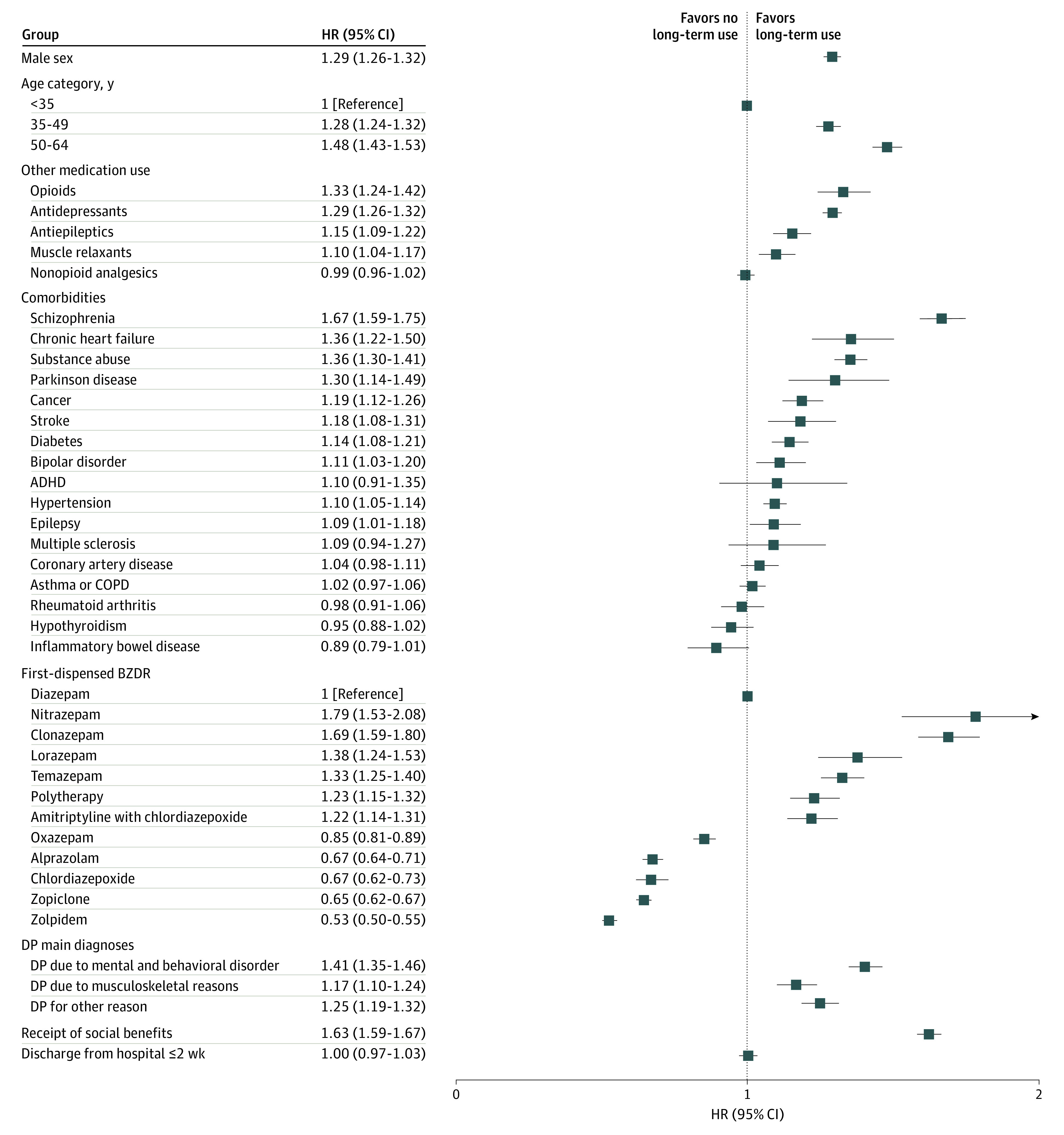
Factors Associated With Long-term Benzodiazepine and Related Drug (BZDR) Use Compared With Short-term Use Among Persons Younger Than 65 Years at BZDR Treatment Initiation The adjusted Cox proportional hazards model is shown for all factors. ADHD indicates attention-deficit/hyperactivity disorder; COPD, chronic obstructive pulmonary disease; DP, disability pension; and HR, hazard ratio.

The adjusted model for characteristics associated with long-term BZDR treatment in the younger subcohort identified several sociodemographic factors and comorbid conditions (Figure 2). These were male sex; older working age; use of opioids, antidepressants, antiepileptics, and/or muscle relaxants; and comorbidities including schizophrenia, substance abuse, chronic heart failure, and Parkinson disease. Having a disability pension and receiving social benefits were also associated with long-term use. In terms of initiating BZDRs compared with diazepam initiation, nitrazepam, clonazepam, lorazepam, temazepam, polytherapy, and an amitriptyline-chlordiazepoxide combination were positively associated with long-term use, whereas oxazepam, alprazolam, chlordiazepoxide alone, zopiclone, and zolpidem were inversely associated with long-term use.

**Figure 3.**
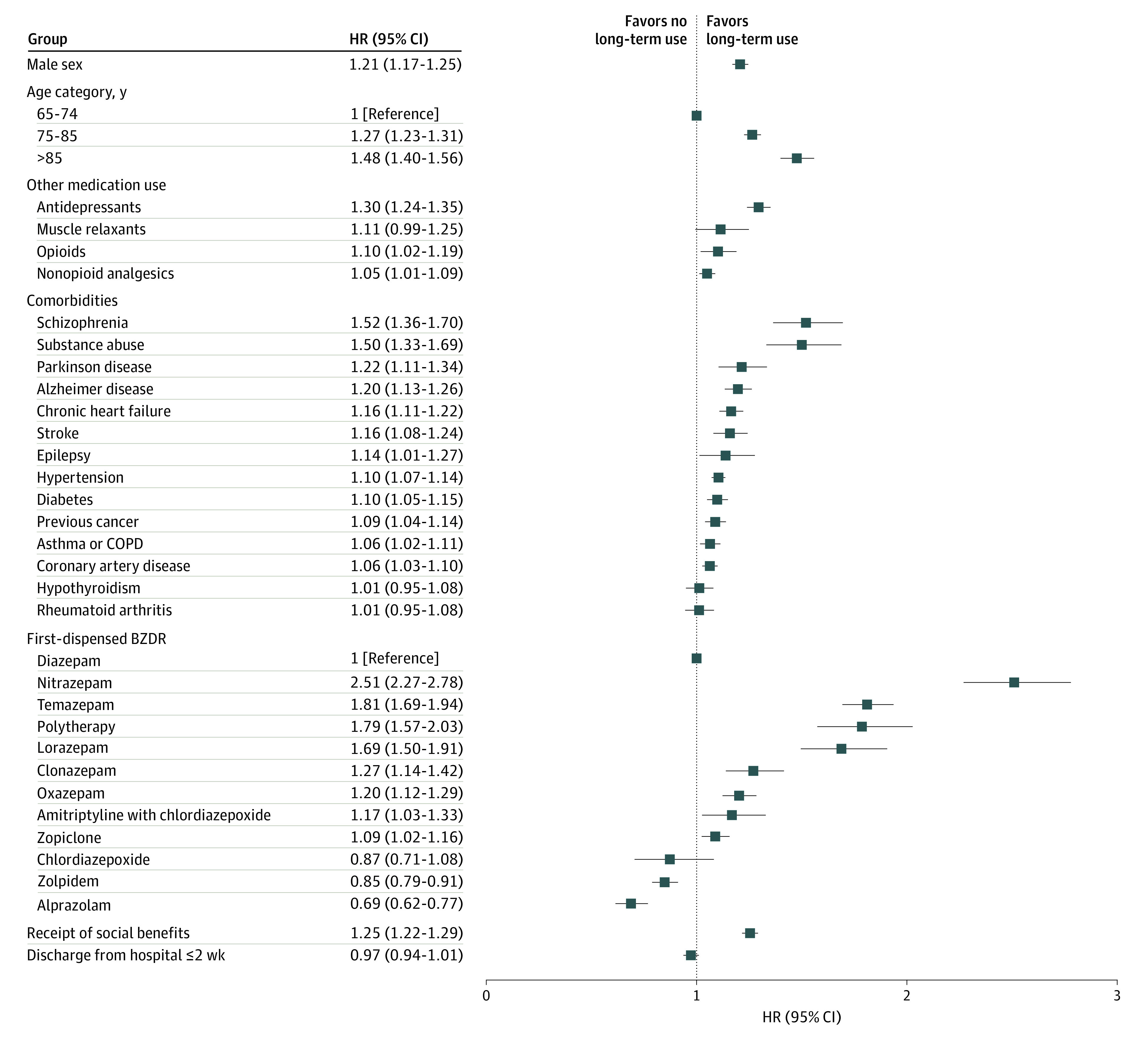
Factors Associated With Long-term Benzodiazepine and Related Drug (BZDR) Use Compared With Short-term Use Among Persons Aged 65 Years or Older at BZDR Treatment Initiation The adjusted Cox proportional hazards model is shown for all factors. COPD indicates chronic obstructive pulmonary disease; HR, hazard ratio.

The adjusted model for the older subcohort identified characteristics associated with long-term treatment as somewhat similar to those in the younger subcohort (Figure 3). These were male sex, older age, use of antidepressants or opioids, receipt of social benefits, and comorbidities including schizophrenia, substance abuse, Parkinson disease, and Alzheimer disease, with almost all other comorbid conditions also showing a positive association with long-term treatment (HRs 1.06-1.16). When compared with initiation of treatment with diazepam, initiation with nitrazepam, temazepam, polytherapy, lorazepam, clonazepam, oxazepam, amitriptyline-chlordiazepoxide, and zopiclone were positively associated with long-term use, whereas zolpidem and alprazolam were inversely associated with long-term use. Characteristics associated with long-term BZDR use in the total cohort are reported in Table 2, and the adjusted model is shown in eFigure 3 in the Supplement.

### Sensitivity Analyses

In sensitivity analyses, with a permissible gap of 365 days, 43 945 BZDR users (33.9%) became long-term users. In the subcohorts, 27 045 persons (28.6%) younger than 65 years and 16 900 (48.2%) of those aged 65 years or older became long-term users by this definition. By restricting the cohort to exclude epilepsy, the proportions of individuals turning to long-term use were similar to the main analyses (39.2% of the full cohort, 33.6% of the younger subcohort, and 54.4% of the older subcohort). Only 109 of 3382 clonazepam treatment initiators used the drug for epilepsy indication, based on reimbursements.

## Discussion

Prescribing guidelines strongly emphasize that BZDR treatment should only be prescribed for a short term (ie, between 4 and 12 weeks, including a discontinuation period).^6,7,8,9,10,11,12,13^ Yet, the results of this study suggest that new BZDR users frequently become long-term users; one-third of persons younger than 65 years and about 55% of persons aged 65 years or older were defined as long-term users, and a substantial proportion developed long-term use quickly, without having any gaps in use after initiation. Use of clonazepam, lorazepam, temazepam, and polytherapy at initiation of treatment was associated with long-term treatment when compared with diazepam, whereas an inverse association with long-term treatment was found for alprazolam and zolpidem. Becoming a long-term user was associated with male sex, older age, receipt of social benefits, psychiatric comorbidities, substance abuse, and use of opioids and/or antidepressants.

This study’s findings suggest that the incidence of subsequent long-term use after BZDR initiation is substantially high. A systematic review of previous studies found that long-term use (defined as ≥6 months of use) was observed in 6% to 15% of all people who used BZDRs and in up to 50% of older persons who used them.^20^ Previous studies have mainly assessed long-term BZDR use in prevalent users, whereas this study population included only incident BZDR users. Previous studies assessing new BZDR users have found that the incidence of long-term use is 11.8% in the general population,^33^ 9% among working-aged persons (requiring longer use periods of ≥8 months without gaps),^34^ 26% among older adults,^35^ 22% among persons with bipolar disorder,^29^ and 30% among older persons with Alzheimer disease.^36^ All of these estimates are lower than the 39.4% incidence reported in this study. Methodological differences, such as whether or not gaps were allowed in drug use, exist among these studies. However, we also found that 22% of people who used BZDRs developed long-term use within the first 6 months (without having any gaps in use). This estimate is similar to a previous Japanese study in which 20% of BZDR initiators used the drug for 6 months or longer.^37^

The high incidence of long-term BZDR use may reflect a lack of effective and safe alternatives to these drugs. Selective serotonin reuptake inhibitors and serotonin-norepinephrine reuptake inhibitors may be used for treatment of anxiety disorders because they are considered to have a more favorable risk-benefit ratio than BZDRs,^38^ although discontinuation or withdrawal syndrome associated with discontinued use of these drugs is currently debated.^39^ The majority of previous studies have consistently shown that the most common long-term BZDR treatment pattern is with a low and steady dosage, without dose escalation.^20^ For this reason, some clinicians insist that there is a small proportion of working-aged long-term BZDR users who experience a favorable risk-benefit ratio and relatively few adverse effects.^38^ Further research is needed for the assessment of risks and benefits of BZDRs and their alternatives for these patients. At the same time, the adverse events associated with long-term BZDR treatment, especially among older individuals, such as increased risk of falls^22^ and fractures,^23,24^ are a real problem. Owing to aging-related changes in the distribution and elimination of drugs,^40^ the elimination half-life of BZDRs is prolonged by aging, which predisposes older individuals to adverse effects and events. Older individuals also have a higher sensitivity to the pharmacological action of benzodiazepines, which is not explained by pharmacokinetic factors such as plasma concentrations, half-life, or volume of distribution.^41^ These pharmacodynamic changes may present as pronounced sedation and sensitivity to cognitive effects, which should be kept in mind in clinical decision-making. Possibly lower risk of misuse of BZDRs in older persons should be balanced with the substantial risk of becoming long-term users, as shown in this study. Although some BZDRs were associated with a lower risk of long-term use, the risk reported was relative to that of diazepam; thus, the lower HRs for some BZDRs should not be interpreted as their being safer options in absolute terms when prescribing these medications.

The clinical choice of the initial BZDR was associated with a differential risk for long-term use. Clonazepam was associated with a higher risk for development of long-term use, which is consistent with previous prevalence studies.^28,29^ Clonazepam is a high-potency benzodiazepine with a long elimination half-life,^42^ and it was used mainly for nonepilepsy indications in this study. However, its high potency may be associated with escalated development of tolerance and related adverse events. Use of clonazepam should be carefully considered, as its abuse liability has been documented.^43^ Also, starting treatment with lorazepam, temazepam, or polytherapy was associated with a higher risk for long-term use, whereas the drugs most often prescribed, oxazepam and Z-drugs, were associated with the lowest risk for long-term use, supporting the rationale for current BZDR treatment guidelines and recommendations.

This study’s finding that alprazolam was associated with a lower risk for long-term use was somewhat surprising, as alprazolam has been associated with increased risk of long-term use in previous studies^29,33^ and is suspected to be more likely to cause physical dependence because of its high potency and short half-life.^44,45^ It is possible that alprazolam was prescribed selectively or restrictively in the whole study population owing to concerns of dependence and was avoided in persons judged to have a high liability for dependence. This selection may also apply to other specific BZDRs, and thus, the association with relative safety does not imply that any BZDR would be resistant to misuse or abuse.

We identified multiple factors associated with development of long-term use, including male sex, older age, receipt of social benefits, psychiatric comorbidities, and substance abuse. Most of these factors have also been reported in previous studies,^20,26,29,34,36,46^ with some controversies, eg, regarding sex.^20,29,34,36,46^ These findings can be useful in clinical decision-making by identifying more precisely those patients who might be at risk of developing long-term use of BZDRs.

### Strengths and Limitations

This study has strengths. It included a large, nationwide cohort of adults initiating BZDR use, which ensured good generalizability. The new-user design with a 2-year washout period was used to define incident users, and the long follow-up time enabled identification of persons who became long-term users somewhat later after the first use. We used the recommended definition of long-term use^20,47^ as 6 months or longer and modeled duration of drug use with the validated PRE2DUP method.^32,48^ Our classification of BZDRs included the whole range of benzodiazepine-related substances as well as clonazepam, which is coded as an antiepileptic in the ATC classification, and a combination product containing chlordiazepoxide and amitriptyline.

This study also has limitations. Some (but not all) smaller packages of BZDRs were not reimbursed and thus were not recorded in the data. It is possible that some persons initiated use with a smaller, nonreimbursed package and continued with a larger, reimbursed package; therefore, this study’s estimates of long-term use would be conservative.

The study cohort was restricted to community-dwelling persons in a single country with a relatively homogeneous population, which may limit generalizability. Some new BZDR users may have previously used BZDRs before the BZDR-free period of 2 years. Although a range of comorbid conditions was derived from registers, these data sources have inherent limitations. Register-based data do not indicate whether dispensed drugs have actually been used^49^; this, however, may be a lesser problem than recreational use. The Care Register for Health Care data were based on inpatient care only, and thus, diagnoses may have represented only the most severe cases. For many diagnoses, data from the special reimbursement register were also used, and the register also provides diagnoses from primary care. However, register-based data lack information on severity of diseases and symptoms as well as, for example, smoking, alcohol use, and nutrition, and consequent residual confounding may still exist. Choices made with respect to drugs and drug forms were aimed at excluding BZDR use for epilepsy indication; thus, the results should only be interpreted to nonepilepsy indications. In addition, this study lacked data on whether BZDR use was initiated during hospital care.

## Conclusions

Although BZDRs are a useful medication class in the treatment of various psychiatric and neurological disorders, the known adverse effects related to long-term BZDR treatment are still a frequent challenge for the prescribing clinician. In this study, the proportion of BZDR initiators who developed long-term use was high, especially in the older age group, and was also associated with the initial choice of specific BZDR drug. These results are valuable for optimization of BZDR treatment in clinical practice, for algorithms designed for knowledge-based treatment decisions, and in the early consideration of potential clinical and sociodemographic risk factors when prescribing BZDRs for the first time.

## Notes

Supplement. **eAppendix.** From Prescriptions to Drug Use Periods (PRE2DUP) Method **eTable 1.** Definitions for Exposures and Covariates **eTable 2.** Characteristics of Long-term Benzodiazepine and Related Drug (BZDR) Users Compared With Short-term users and Unadjusted HRs for Persons <65 Years at BZDR Initiation **eTable 3.** Characteristics of Long-term Benzodiazepine and Related Drug (BZDR) Users Compared With Short-term Users and Unadjusted HRs for Persons ≥65 Years at BZDR Initiation **eFigure 1.** Study Design and Definitions for Long-term Benzodiazepine and Related Drug (BZDR) Use **eFigure 2.** Time in Years to Initiation of Long-term BZDR Use **eFigure 3.** Factors Associated With Long-term Benzodiazepine and Related Drug (BZDR) Use Compared With Short-term Use in the Entire Cohort
