# Supplementary material for: Incidence of and Characteristics Associated With Long-term Benzodiazepine Use in Finland
Source: JAMA Netw Open. 2020 Oct 29;3(10):e2019029. doi: 10.1001/jamanetworkopen.2020.19029 (PMC7596584; doi:10.1001/jamanetworkopen.2020.19029)
Supplement: Supplement. — eAppendix. From Prescriptions to Drug Use Periods (PRE2DUP) Method eTable 1. Definitions for Exposures and Covariates eTable 2. Characteristics of Long-term Benzodiazepine and Related Drug (BZDR) Users Compared With Short-term users and Unadjusted HRs for Persons <65 Years at BZDR Initiation eTable 3. Characteristics of Long-term Benzodiazepine and Related Drug (BZDR) Users Compared With Short-term Users and Unadjusted HRs for Persons ≥65 Years at BZDR Initiation eFigure 1. Study Design and Definitions for Long-term Benzodiazepine and Related Drug (BZDR) Use eFigure 2. Time in Years to Initiation of Long-term BZDR Use eFigure 3. Factors Associated With Long-term Benzodiazepine and Related Drug (BZDR) Use Compared With Short-term Use in the Entire Cohort [file jamanetwopen-e2019029-s001.pdf]

Supplementary Online Content

Taipale H, Särkilä H, Tanskanen A, et al. Incidence of and characteristics associated with long-term benzodiazepine use in Finland. *JAMA Netw Open*. 2020;3(10):e2019029. doi:10.1001/jamanetworkopen.2020.19029

eAppendix. From Prescriptions to Drug Use Periods (PRE2DUP) Method

eTable 1. Definitions for Exposures and Covariates

eTable 2. Characteristics of Long-term Benzodiazepine and Related Drug (BZDR) Users Compared With Short-term Users and Unadjusted HRs for Persons <65 Years at BZDR Initiation

eTable 3. Characteristics of Long-term Benzodiazepine and Related Drug (BZDR) Users Compared With Short-term Users and Unadjusted HRs for Persons ≥65 Years at BZDR Initiation

eFigure 1. Study Design and Definitions for Long-term Benzodiazepine and Related Drug (BZDR) Use

eFigure 2. Time in Years to Initiation of Long-term BZDR Use

eFigure 3. Factors Associated With Long-term Benzodiazepine and Related Drug (BZDR) Use Compared With Short-term Use in the Entire Cohort

This supplementary material has been provided by the authors to give readers additional information about their work.

© 2020 Taipale H et al. *JAMA Network Open*.

eAppendix.

From Prescriptions to Drug use Periods (PRE2DUP) method

Duration of continuous benzodiazepine and related drug (BZDR) use was defined with 'From Prescriptions to Drug Use Periods' (PRE2DUP) model. The method is based on calculation of sliding averages of daily dose and dose is modelled according to individual purchasing behavior for each person and ATC code. Joining of purchases into drug use periods (when drug use started and ended) are controlled with expert-defined parameters for each drug package and method also takes into account on regularity of purchases, stockpiling of drugs and hospital care periods when drugs are provided by the health care institution. Duration for single purchases (i.e. when person has only purchase of a drug, or other purchases are too far for joining, such as years apart) is assigned according to the most common refill length calculated from the study population. As age range in this study is wide and age may impact on typical dose and use pattern of BZDRs (e.g. due to aging-related changes in pharmacokinetics and pharmacodynamics), duration for single purchases was modelled separately for younger sub-cohort (<65 years) and older sub-cohort (≥65 years).

Detailed description of PRE2DUP method has been previously published:

Tanskanen A, Taipale H, Koponen M, et al. From prescription drug purchases to drug use periods – a second generation method (PRE2DUP). *BMC Med Inform Decis Mak*. 2015;15(1):21.

© 2020 Taipale H et al. *JAMA Network Open*.

eTable 1. Definitions for exposures and covariates

**EXPOSURE**			
**Included exposure categories**	**Included specific drugs** (all specific drugs marked in Finland of the included exposure categories were included and listed here)	**Exceptions and clarifications**	**Data source**
N03AE01 (clonazepam)	N03AE01 clonazepam	-	PR
N05BA (Anxiolytics, benzodiazepine derivatives)	N05BA01 diazepam<br>N05BA02 chlordiazepoxide<br>N05BA04 oxazepam<br>N06BA06 lorazepam<br>N05BA12 alprazolam	N05BA09 clobazam excluded as it is only used for treatment of epilepsy.<br>Only orally administered dosage forms were included with the exception of oral suspensions (also mainly used for epilepsy).	PR
N05CD (Hypnotics and sedatives, benzodiazepine derivatives)	N05CD02 nitrazepam<br>N05CD07 temazepam	N05CD05 triazolam and N05CD08 midazolam are not reimbursed in Finland (rarely used and subjected to closer monitoring than other BZDRs)	PR
N05CF (Hypnotics and sedatives, benzodiazepine related drugs)	N05CF01 zopiclone<br>N05CF02 zolpidem	N05CF03 zaleplon not reimbursed in Finland	PR
N06CA01 (Amitriptyline and psycholeptics)	N06CA01 combination products marketed in Finland include amitriptyline and chlordiazepoxide (5 or 10 mg)	Other combinations of amitriptyline (i.e. including perphenazine) excluded.	PR
**COVARIATES**			
**Covariate**	**Classification**	**Measurement period**	**Data source**
Age, years	18-34, 35-49, 50-64, 65-74, 75-84, ≥85	At the BZDR initiation (=start of follow-up)	PR
**Comorbidities**	**ICD-10/<br>special reimbursement code/<br>ATC code**		
Hypertension	I10-I15/<br>205	Diagnosed during two years (HDR) or ever (DPR, SR) before start of follow-up	HDR, DPR, SR
Coronary artery disease	I20-I25/<br>206, 213, 280	Diagnosed during two years (HDR) or ever (DPR, SR) before start of follow-up	HDR, DPR, SR

© 2020 Taipale H et al. *JAMA Network Open*.

Chronic heart failure	I42, I43, I50, I11.0/<br>201, 283, 289	Diagnosed during two years (HDR) or ever (DPR, SR) before start of follow-up	HDR, DPR, SR
Stroke	I60-I64, I69	Diagnosed during two years (HDR) or ever (DPR) before start of follow-up	HDR, DPR
Diabetes	E10-E14/<br>103, 171, 215, 285, 371	Diagnosed during two years (HDR) or ever (DPR, SR) before start of follow-up	HDR, DPR, SR
Asthma/COPD	J44-J46<br>/203	Diagnosed during two years (HDR) or ever (DPR, SR) before start of follow-up	HDR, DPR, SR
Rheumatoid arthritis	M05, M06, M45/<br>202	Diagnosed during two years (HDR) or ever (DPR, SR) before start of follow-up	HDR, DPR, SR
Cancer	C00-C97/<br>115, 116, 117, 128, 130, 180, 184, 185, 189, 311, 312, 316	Diagnosed during two years (HDR) or ever (DPR, SR) before start of follow-up	HDR, DPR, SR
Hypothyroidism	104	Diagnosed ever before start of follow-up	SR
IBD	208	Diagnosed ever before start of follow-up	SR
Parkinson disease	G20/<br>110/<br>N04B	Diagnosed during two years (HDR) or ever (DPR, SR), or drug use (PR) during two years before start of follow-up	HDR, DPR, SR, PR
Alzheimer's disease	G30, F00-F03/<br>307/<br>N06D	Diagnosed during two years (HDR) or ever (DPR, SR), or drug use (PR) during two years before start of follow-up	HDR, DPR, SR, PR
MS disease	G35/<br>109, 157, 303	Diagnosed during two years (HDR) or ever (DPR, SR) before start of follow-up	HDR, DPR, SR
Epilepsy	111	Diagnosed ever before start of follow-up	SR
Schizophrenia	F20-F29/<br>112 with schizophrenia diagnoses	Diagnosed during two years (HDR) or ever (DPR, SR) before start of follow-up	HDR, DPR, SR
Bipolar disorder	F30, F31/<br>112 with bipolar disorder diagnoses	Diagnosed during two years (HDR) or ever (DPR, SR) before start of follow-up	HDR, DPR, SR

© 2020 Taipale H et al. *JAMA Network Open*.

Substance abuse	K70, K86.0, F10-F19/<br>[no special reimbursement codes]/<br>N07BB, N07BC01, N07BC02, N07BC51	Diagnosed during two years (HDR) or ever (DPR), or drug use (PR) during two years before start of follow-up	HDR, DPR, PR
ADHD	F90/<br>[no special reimbursement codes]/<br>N06BA	Diagnosed during two years (HDR) or ever (DPR), or drug use (PR) during two years before start of follow-up	HDR, DPR, PR
**Medication use**	**ATC codes**		
Antidepressants	N06A	Within 30 days prior to the start of follow-up	PR
Antipsychotics	N05A excluding lithium N05AN01	Within 30 days prior to the start of follow-up	PR
Antiepileptics	N03A	Within 30 days prior to the start of follow-up	PR
Opioids	N02A	Within 30 days prior to the start of follow-up	PR
Non-opioid analgesics	M01A (NSAIDs), excluding glucosamine, N02BE01 paracetamol	Within 30 days prior to the start of follow-up	PR
Muscle relaxants	M03	Within 30 days prior to the start of follow-up	PR
**Other factors**			
Discharged from hospital ≤2 weeks	Discharge from hospital care	Within 14 days prior to the start of follow-up	HDR
Receipt of social benefits	Based on basic social assistance, labour market subsidy, basic unemployment allowance, national pension and study grants.	During the year 2005	
Receipt of childcare benefits	Based on maternity allowance, paternity allowance and child home care allowance.	During the year 2005	
Disability pension	Categorized as:<br>-no DP<br>-DP due to mental and behavioural disorders (ICD-10 F00-F99)<br>-DP due to musculoskeletal reasons (ICD-10 M00-M99)<br>-DP due to other reasons (excluding F and M categories)	Ever before start of follow-up (ongoing at the follow-up)	DPR
Choice of covariates for the adjusted model: variables with a strong correlation to one or several other variables, and variables with the cell count <200 were removed. Thus, the following factors were excluded from younger subpopulation (<65 years) model: Alzheimer's disease, childcare (correlated with youngest age group), and antipsychotic use (correlated with schizophrenia), and the following factors from older subpopulation (≥65 years) model: inflammatory bowel disease (IBD), MS disease, bipolar disorder, ADHD, antipsychotic use (correlated with schizophrenia) and antiepileptic use (correlated with epilepsy).			

© 2020 Taipale H et al. *JAMA Network Open*.

Abbreviations: AD, Alzheimer’s disease; ATC, Anatomical Therapeutic Chemical; COPD, chronic obstructive pulmonary disease; HDR, Hospital Discharge Register; ICD, International Classification of Diseases; NOMESCO: The Nordic Medico-Statistical Committee; PR, Prescription Register; SR, Special Reimbursement Register. IBD: inflammatory bowel disease, MS: multiple sclerosis, ADHD: attention-deficit hyperactivity disorder, DPR: Disability Pension Register

© 2020 Taipale H et al. *JAMA Network Open*.

eTable 2. Characteristics of long-term benzodiazepine and related drug (BZDR) users compared with short-term users and unadjusted HRs for persons <65 years at BZDR initiation.					
	Short-term users		Long-term users		Unadjusted HR (95% CI)
	N	%	N	%	
Age categories					
18-34	16892	26.95	6439	20.12	reference
35-49	22667	36.16	10907	34.09	1.12 (1.09-1.16)
50-64	23119	36.89	14650	45.79	1.35 (1.32-1.40)
Male gender	24438	38.99	15145	47.33	1.39 (1.36-1.42)
**First dispensed BZDR**					
diazepam	3817	6.09	3076	9.61	reference
clonazepam	1049	1.67	1488	4.65	1.63 (1.53-1.73)
chlordiazepoxide	1117	1.78	691	2.16	0.80 (0.73-0.86)
oxazepam	9465	15.10	5437	16.99	0.75 (0.71-0.78)
lorazepam	267	0.43	407	1.27	1.76 (1.58-1.95)
alprazolam	6666	10.64	2598	8.12	0.53 (0.50-0.55)
nitrazepam	92	0.15	171	0.53	1.84 (1.58-2.15)
temazepam	1457	2.32	1961	6.13	1.40 (1.32-1.48)
zopiclone	20946	33.42	9230	28.85	0.53 (0.51-0.55)
zolpidem	15146	24.16	4842	15.13	0.39 (0.38-0.41)
amitriptyline + chlordiazepoxide	1613	2.57	1029	3.22	0.94 (0.87-1.00)
polytherapy	1043	1.66	1066	3.33	1.16 (1.09-1.25)
**Other medication use** (within 30 days prior to BZDR initiation)					
Antipsychotics	2408	3.84	4000	12.5	2.92 (2.82-3.02)
Antidepressant	13250	21.14	8974	28.05	1.42 (1.38-1.45)
Antiepileptics	1692	2.70	1872	5.85	2.03 (1.94-2.12)
Opioids	956	1.53	928	2.9	1.83 (1.71-1.95)
Non-opioid analgesics	10230	16.32	5357	16.74	1.03 (1.00-1.06)
Muscle relaxants	2133	3.4	1254	3.92	1.12 (1.06-1.18)
**Comorbidities**					
Hypertension	5642	9.00	4032	12.6	1.28 (1.24-1.33)
Coronary artery disease	1317	2.10	1119	3.50	1.44 (1.36-1.53)
Chronic heart failure	319	0.51	384	1.20	2.03 (1.83-2.24)
Stroke	372	0.59	427	1.33	2.00 (1.82-2.20)
Diabetes	1553	2.48	1415	4.42	1.64 (1.55-1.72)
Rheumatoid arthritis	1259	2.01	701	2.19	1.04 (0.96-1.12)
Asthma/COPD	3754	5.99	2123	6.64	1.08 (1.03-1.12)
Cancer	1805	2.88	1202	3.76	1.28 (1.21-1.36)
Hypothyroidism	1206	1.92	664	2.08	0.98 (0.91-1.06)
IBD	564	0.90	277	0.87	0.92 (0.81-1.03)
Parkinson disease	140	0.22	229	0.72	2.64 (2.32-3.01)
Alzheimer's disease	56	0.09	96	0.30	2.92 (2.39-3.56)
MS disease	203	0.32	173	0.54	1.47 (1.26-1.70)
Epilepsy	917	1.46	743	2.32	1.57 (1.46-1.68)

© 2020 Taipale H et al. *JAMA Network Open*.

Schizophrenia	1448	2.31	2341	7.32	2.78 (2.67-2.90)
Bipolar disorder	657	1.05	781	2.44	1.96 (1.83-2.11)
Substance abuse	2574	4.11	2967	9.27	2.13 (2.05-2.21)
ADHD	111	0.18	99	0.31	1.55 (1.28-1.89)
**Other factors**					
Discharge from hospital ≤2 weeks	8604	13.73	5983	18.70	1.49 (1.45-1.53)
Receipt of social benefits	15270	24.36	12351	38.60	1.88 (1.84-1.92)
Receipt of childcare benefits during previous year	10436	16.65	2629	8.22	0.55 (0.53-0.58)
**Disability pension (DP)**					
No DP	57059	91.04	26025	81.34	reference
DP due mental and behavioral disorder	2205	3.52	2961	9.25	2.31 (2.22-2.40)
DP due to musculoskeletal reasons	1665	2.66	1253	3.92	1.44 (1.36-1.53)
DP due to other reason	1749	2.79	1757	5.49	1.90 (1.81-1.99)
COPD: chronic obstructive pulmonary disease, IBD: inflammatory bowel disease, MS: multiple sclerosis, ADHD: attention-deficit hyperactivity disorder, DP: disability pension.					

© 2020 Taipale H et al. *JAMA Network Open*.

eTable 3. Characteristics of long-term benzodiazepine and related drug (BZDR) users compared with short-term users and unadjusted HRs for persons ≥65 years at BZDR initiation.					
	Short-term users		Long-term users		Unadjusted HR (95% CI)
	N	%	N	%	
Age categories					
65-74	8743	54.80	8643	45.24	reference
75-85	6123	38.38	8687	45.47	1.43 (1.38-1.47)
>85	1089	6.83	1773	9.28	1.86 (1.77-1.96)
Male gender	5297	33.20	6835	35.78	1.15 (1.12-1.19)
**First dispensed BZDR**					
diazepam	1286	8.06	1203	6.30	reference
clonazepam	387	2.43	458	2.40	1.28 (1.15-1.42)
chlordiazepoxide	123	0.77	91	0.48	0.90 (0.73-1.11)
oxazepam	2645	16.58	3031	15.87	1.23 (1.15-1.31)
lorazepam	190	1.19	353	1.85	1.99 (1.77-2.24)
alprazolam	758	4.75	415	2.17	0.65 (0.58-0.73)
nitrazepam	124	0.78	529	2.77	2.66 (2.40-2.94)
temazepam	1364	8.55	3038	15.90	1.97 (1.85-2.11)
zopiclone	6378	39.97	7497	39.25	1.08 (1.02-1.15)
zolpidem	2231	13.98	1894	9.91	0.79 (0.74-0.85)
amitriptyline + chlordiazepoxide	333	2.09	286	1.50	1.07 (0.94-1.22)
polytherapy	136	0.85	308	1.61	1.98 (1.75-2.24)
**Other medication use** (within 30 days prior to BZDR initiation)					
Antipsychotic	720	4.51	1453	7.61	1.89 (1.79-1.99)
Antidepressant	1554	9.74	2487	13.02	1.42 (1.36-1.49)
Antiepileptics	431	2.70	597	3.13	1.24 (1.14-1.34)
Opioids	516	3.23	697	3.65	1.27 (1.18-1.37)
Non-opioid analgesic	3036	19.03	3818	19.99	1.09 (1.06-1.13)
Muscle relaxant	243	1.52	304	1.59	1.04 (0.93-1.17)
**Comorbidities**					
Hypertension	5804	36.38	7828	40.98	1.16 (1.13-1.20)
Coronary artery disease	3548	22.24	5002	26.18	1.22 (1.18-1.26)
Chronic heart failure	1267	7.94	2121	11.10	1.48 (1.42-1.55)
Stroke	559	3.50	846	4.43	1.43 (1.34-1.53)
Diabetes	1595	10.00	2276	11.91	1.26 (1.20-1.31)
Rheumatoid arthritis	752	4.71	897	4.70	1.02 (0.95-1.09)
Asthma/COPD	1642	10.29	2122	11.11	1.09 (1.05-1.14)
Cancer	1797	11.26	2155	11.28	1.10 (1.05-1.15)
Hypothyroidism	844	5.29	1007	5.27	0.98 (0.92-1.05)
IBD	92	0.58	103	0.54	0.93 (0.77-1.13)
Parkinson disease	307	1.92	453	2.37	1.41 (1.28-1.55)
Alzheimer's disease	1028	6.44	1629	8.53	1.64 (1.56-1.73)
MS disease	18	0.11	22	0.12	1.15 (0.75-1.74)
Epilepsy	209	1.31	297	1.55	1.22 (1.09-1.37)

© 2020 Taipale H et al. *JAMA Network Open*.

Schizophrenia	155	0.97	347	1.82	1.85 (1.67-2.06)
Bipolar disorder	30	0.19	58	0.30	1.64 (1.27-2.13)
Substance abuse	159	1.00	287	1.50	1.52 (1.36-1.71)
ADHD	1	0.01	1	0.01	1.00 (0.14-6.93)
**Other factors**					
Discharge from hospital ≤2 weeks	3129	19.61	3627	18.99	1.15 (1.11-1.19)
Receipt of social benefits	7971	49.96	11121	58.22	1.34 (1.30-1.38)

COPD: chronic obstructive pulmonary disease, IBD: inflammatory bowel disease, MS: multiple sclerosis, ADHD: attention-deficit hyperactivity disorder, DP: disability pension.

© 2020 Taipale H et al. *JAMA Network Open*.

eFigure 1. Study design and definitions for long-term benzodiazepine and related drug (BZDR) use.

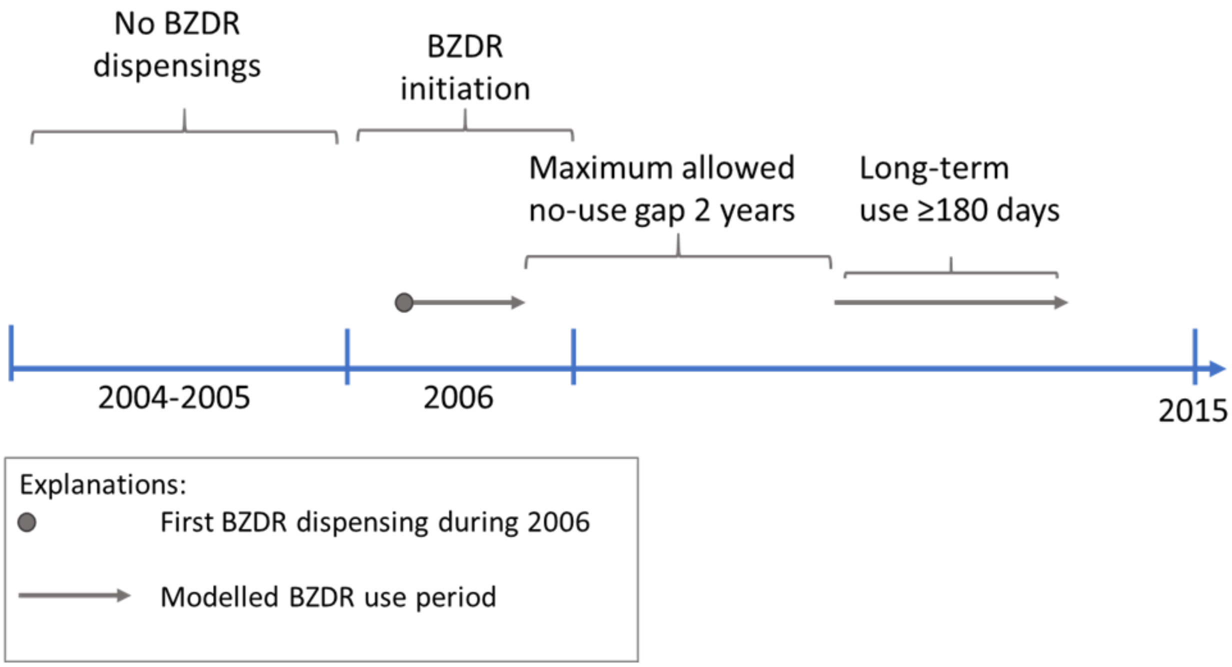

© 2020 Taipale H et al. *JAMA Network Open*.

eFigure 2. Time in years to initiation of long-term BZDR use. A) The whole cohort (N=129,732), B) stratified by age; younger referring to those aged <65 years (N=94,674, in blue color) and older to those aged ≥65 years (N=55,091, in green color). N of persons at risk is presented every second year in the lower part of the figure.A.

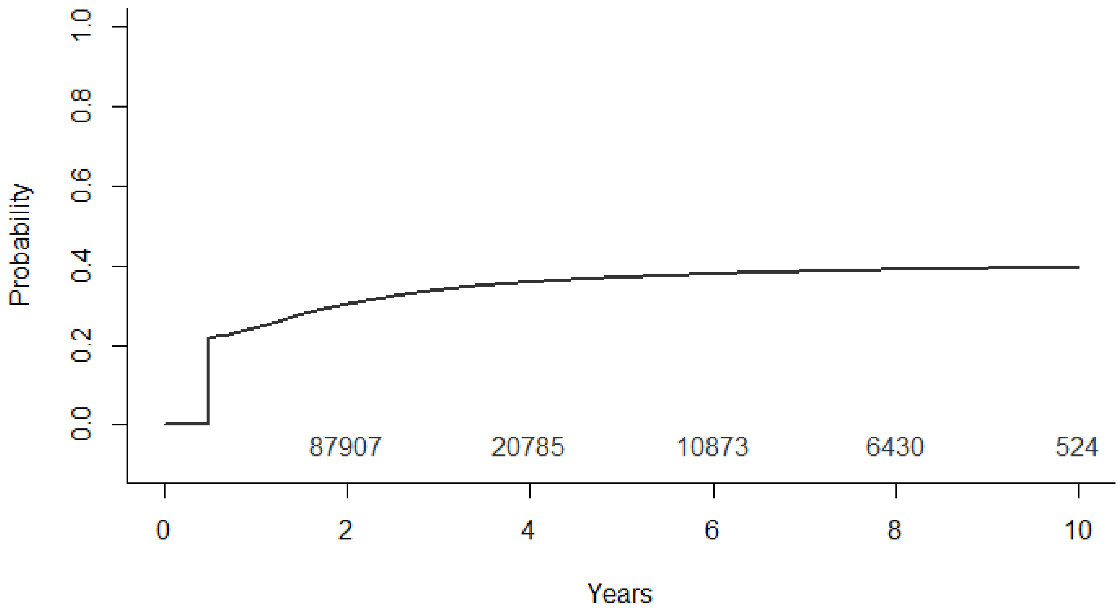

B.

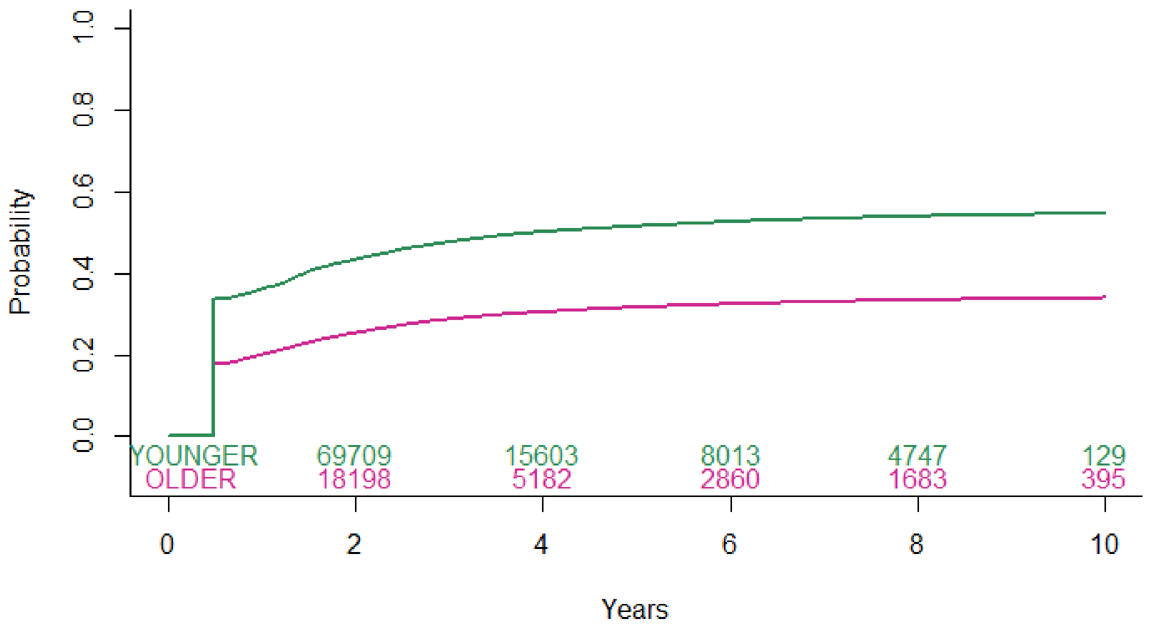

© 2020 Taipale H et al. *JAMA Network Open*.

eFigure 3. Factors associated with long-term benzodiazepine and related drug (BZDR) use compared with short-term use in the entire cohort. Adjusted Cox model (for all factors shown in figure), Hazard Ratios (HR) with 95% Confidence Intervals (CIs). **MS: multiple sclerosis, COPD: chronic obstructive pulmonary disease, IBD: inflammatory bowel disease, ADHD: attention deficit hyperactivity disorder.**

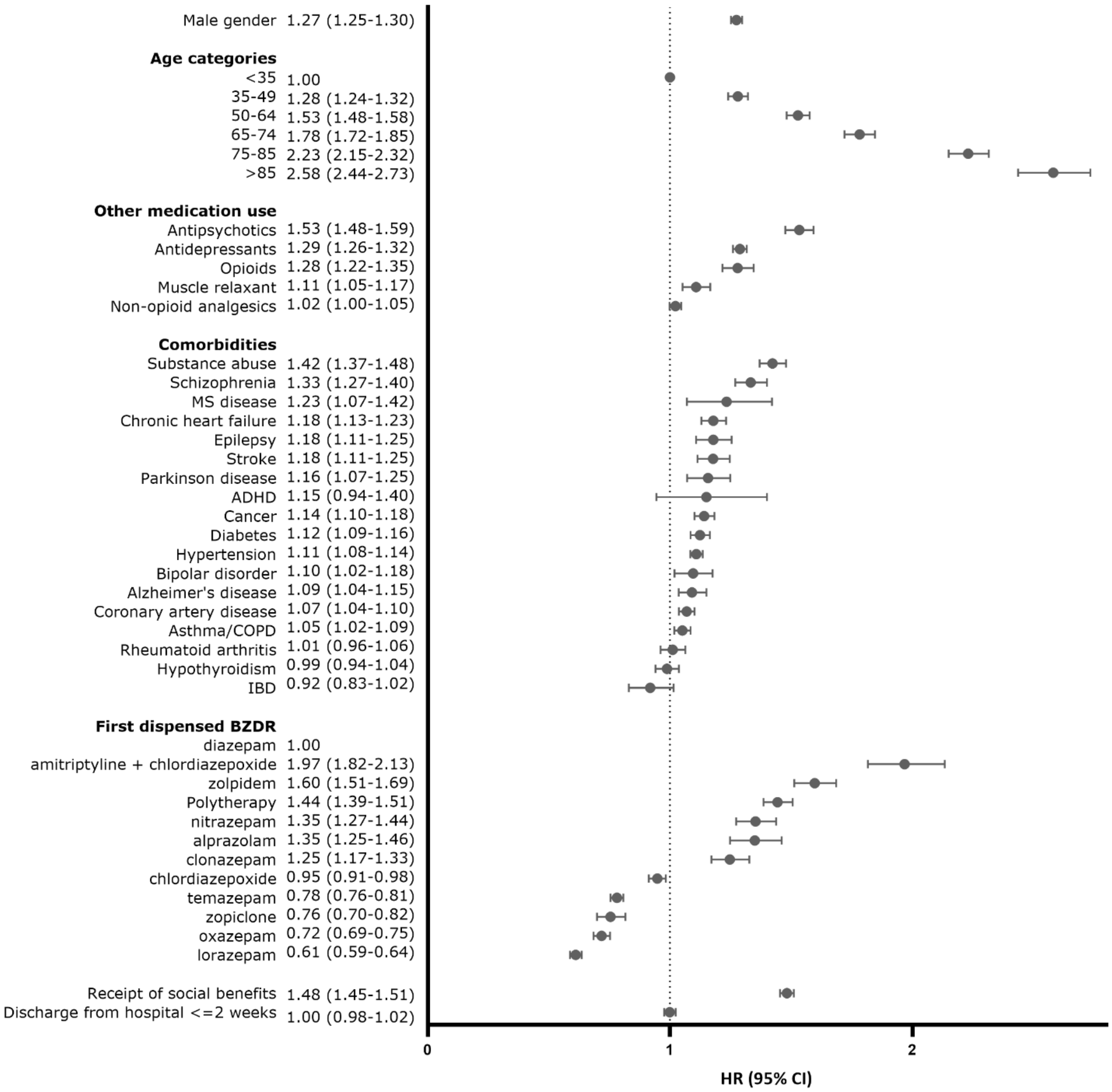

© 2020 Taipale H et al. *JAMA Network Open*.
